# Molecular mechanisms of microRNA-216a during tumor progression

**DOI:** 10.1186/s12935-023-02865-2

**Published:** 2023-02-05

**Authors:** Amir Abbas Hamidi, Negin Taghehchian, Amir Sadra Zangouei, Iman Akhlaghipour, Amirhosein Maharati, Zahra Basirat, Meysam Moghbeli

**Affiliations:** 1 Student Research Committee, Faculty of Medicine, Mashhad University of Medical Sciences, Mashhad, Iran; 2 Medical Genetics Research Center, Mashhad University of Medical Sciences, Mashhad, Iran; 3 Department of Medical Genetics and Molecular Medicine, School of Medicine, Mashhad University of Medical Sciences, Mashhad, Iran

**Keywords:** MicroRNA-216a, Diagnosis, Prognosis, Cancer, Marker, Treatment

## Abstract

MicroRNAs (miRNAs) as the members of non-coding RNAs family are involved in post-transcriptional regulation by translational inhibiting or mRNA degradation. They have a critical role in regulation of cell proliferation and migration. MiRNAs aberrations have been reported in various cancers. Considering the importance of these factors in regulation of cellular processes and their high stability in body fluids, these factors can be suggested as suitable non-invasive markers for the cancer diagnosis. MiR-216a deregulation has been frequently reported in different cancers. Therefore, in the present review we discussed the molecular mechanisms of the miR-216a during tumor progression. It has been reported that miR-216a mainly functioned as a tumor suppressor through the regulation of signaling pathways and transcription factors. This review paves the way to suggest the miR-216a as a probable therapeutic and diagnostic target in cancer patients.

## Background

Cancer is regarded as one of the leading causes of human deaths in the current century. High rate of the cancer mortality and incidence has become a global health challenge [1]. Despite of various therapeutic progresses during the recent decades, there is still a high rate of therapeutic resistance and tumor recurrence among these patients [2]. Therefore, there is an urgent requirement to assess the molecular mechanisms of tumor progression to suggest novel therapeutic targets. Studies over the past two decades have clearly demonstrated that microRNAs (miRNAs) have critical roles in regulation of physiological and pathophysiological cellular processes [3]. MiRNAs are involved in cell proliferation, differentiation, and apoptosis [4, 5]. They may also function as oncogenic or tumor-suppressor, depending on their intracellular roles and expression levels [6, 7]. Moreover, aberrant expression of miRNAs has been associated with therapeutic resistance in cancer that suggests these factors as probable efficient therapeutic targets in tumor cells [8]. Combining miRNA-based therapies with other anticancer treatments is of interest due to the ability of miRNAs to target multiple target genes. Since the function of miRNAs varies according to the tumor type, it is highly desirable to investigate whether miRNA inhibition or replacement therapy can effectively interfere with the signaling pathway associated with therapeutic resistance to enhance the efficacy of anticancer therapy [9, 10]. In addition, early diagnosis can significantly improve treatment outcomes and prolong the survival of cancer patients. Given the high stability of the miRNAs in body fluids and blood, they may represent an excellent set of non-invasive biomarkers for the early cancer diagnosis and prognosis [11]. Accordingly, understanding the regulatory role of these factors during tumor progression can be used for diagnostic and therapeutic purposes [12]. MiR-216a-3p is located on human chromosome 2p16.1 and miR-216 region that contains the miR-216a-3p, miR-216a-5p, miR-216b3p, and miR-216b-5p [13]. MiR-216a participates in various cellular processes and tumor progressions [14–17]. Therefore, in the present review we discussed the molecular mechanisms of miR-216a during tumor progression to introduce that as a reliable diagnostic and prognostic factor in cancer patients (Table 1).

**Table 1:** Molecular targets of miR-216a during tumor progressions

Study	Year	Type	Target	Samples	Function	Clinical Application
Zhao [17]	2020	Cervical cancer	ACTL6A	45 T 45N tissues CaSki, C‐33A, HeLa, and SiHa cell lines	Tumor suppressor	Diagnosis
Hou [28]	2015	Pancreatic cancer	JAK-2	14 T 6N tissues PANC-1, HPDE6c7, BxPC3, CFPAC-1, and Aspc-1 cell lines BALB/c nude mice	Tumor suppressor	Diagnosis
Jin [29]	2018	Ovarian cancer	PTEN	SKOV3 and OVCA433 cell lines	Oncogene	Diagnosis
Cao [34]	2019	Glioma	JAK2	U251 cell line	Tumor suppressor	Diagnosis
Tao [35]	2017	Gastric cancer	JAK2/STAT3	90 T 90N tissues SGC-7901, MGC-803, MKN-28, and BGC-823 cell lines BALB/c nude mice	Tumor suppressor	Diagnosis and prognosis
Wan [41]	2020	Hepatocellular carcinoma	MAPK14	60 T tissues Huh-7, HepG2, and PLC/PRF/5 cell lines BALB/c nude mice	Tumor suppressor	Diagnosis and prognosis
Zhang [44]	2017	Colorectal cancer	KIAA1199/CEMIP	70 T 70N tissues HCT116, SW480, HT29, LOVO and SW620 cell lines NOD/SCID mice	Tumor suppressor	Diagnosis and prognosis
Bai [49]	2019	Hepatocellular carcinoma	PTEN/ Smad7	HCCLM3 cell line	Oncogene	Diagnosis
Cui [50]	2020	Ovarian cancer	PTEN	60 T 60N tissues UWB1.289 cell line	Oncogene	Diagnosis
Liu [51]	2017	Ovarian cancer	PTEN/AKT	87 T 25N tissues SKOV-3, HO-8910, A2780, ES-2, CAOV3, and OVCAR3 cell lines	Oncogene	Diagnosis and prognosis
Wang [53]	2021	Pancreatic cancer	WT1	71 T 71N tissues AsPC-1, BxPC-3, PANC-1, MIA PaCa-2, and SW1990 cell lines BALB/c nude mice	Tumor suppressor	Diagnosis and prognosis
Yang [60]	2018	Prostate cancer	BCL-2	86 T 86N tissues DU145, PC3, LNCaP, and 22Rv1 cell lines	Tumor suppressor	Diagnosis and prognosis
Yu [66]	2020	Non-small cell lung cancer	Wnt	A549, H1975, H1755, H1944, H2087, H358, H661 and H1299 cell lines	Tumor suppressor	Diagnosis
Zhang [71]	2017	glioma	LGR5	15 T 15N tissues U251MG, U87MG, U118, and A172 cell lines	Tumor suppressor	Diagnosis
Lu [92]	2017	Pancreatic cancer	YB-1	72 T 72N tissues Panc-1 and Miapaca-2 cell lines	Tumor suppressor	Diagnosis
Li [95]	2021	Large B-Cell Lymphoma	YBX1	DB, SU-DHL-10, and SU-DHL-4 cell lines	Tumor suppressor	Diagnosis
Zeng [96]	2019	Colorectal cancer	YBX1	70 T 70N tissues LoVo, SW480, HT-29, HCT-116, and Caco-2 cell lines	Tumor suppressor	Diagnosis and prognosis
Song [98]	2019	Gastric cancer	BRD4	36 T 36N tissues AGS, BGC-823, MKN-45, MGC-803, and SCG-7901 cell lines	Tumor suppressor	Diagnosis
Sun [103]	2021	Esophageal cancer	HMBG3	68 T 68N tissues TE-1, TE-9, KYSE30, EC9706 cell lines	Tumor suppressor	Diagnosis
Wang [104]	2019	Hepatocellular carcinoma	KLF12	Hep3B, HepG2, Huh7, SNU449, SK‐hep‐1, and LO2 cell lines BALB/c nude mice	Tumor suppressor	Diagnosis
Qu [105]	2020	Oral squamous cell carcinoma	BCL-2/ KLF-12	86 T 86N tissues SCC9, SCC15, SCC25, CAL27 and Tca8113 cell lines BALB/c nude mice	Tumor suppressor	Diagnosis and prognosis
Pan [109]	2020	Osteosarcoma	SOX5	45 T 45N tissues MG-63, U2OS, 143B cell lines BALB/C nude mice	Tumor suppressor	Diagnosis and prognosis
Zhen [110]	2018	Lung cancer	DANCR	32 T 11N tissues BEAS-2B, NCI-H1299, A549, and NCI-H1975 cell lines Nude mice	Tumor suppressor	Diagnosis and prognosis
Zhu [114]	2018	Cervical cancer	ZEB1	60 T 18N tissues HeLa, CaSki, SiHa, and C33A cell lines	Tumor suppressor	Diagnosis and prognosis
Zhao [115]	2020	Non-Small Cell Lung Cancer	ZEB1	42 T 42N tissues A549, H322, H1299, GLC-82, and SPC-A1 cell lines BALB/c nude mice	Tumor suppressor	Diagnosis and prognosis
Zhang [118]	2018	Gastric cancer	RUNX1	140 T 140N tissues AGS, MKN-45, and HGC-27 cell lines	Oncogene	Diagnosis and prognosis
Zhang [124]	2015	Pancreatic cancer	beclin-1	PANC-1 cell line BALB/c nude mice	Tumor suppressor	Diagnosis
Zhao [125]	2020	Gastric cancer	BCL-2	106 T 106N tissues SGC7901 cell line BALB/c nude mice	Tumor suppressor	Diagnosis and prognosis
Wang [128]	2019	Colorectal cancer	MAP1S	67 T 67N tissues HT-29, HCT-116, SW-480, and SW-62 cell lines	Tumor suppressor	Diagnosis
Zhang [132]	2020	Pancreatic cancer	TPT1/mTORC1	40 T 40N tissues SW1990, PANC1, Capan-2 and BxPC-3 cell lines SCID mice	Tumor suppressor	Diagnosis and prognosis
Zhou [135]	2021	Oral squamous cell carcinoma	BCL2L2	30 T 30N tissues CAL27 and SCC25 cell lines BALB/c nude mice	Tumor suppressor	Diagnosis
Sun [137]	2018	Small cell lung cancer	BCL-2	NCI-H69, NCI-H69AR, NCI-H446, and 16-HBE cell lines BALB/c nude mice	Tumor suppressor	Diagnosis
Ji [144]	2017	osteosarcoma	CDK14	91 T 91N tissues U2OS and 143B cell lines BALB/c nude mice	Tumor suppressor	Diagnosis and prognosis
Lin [148]	2021	Bladder carcinoma	BTG2	21 T 21N tissues EJ, T24, 5637, TCC-SUP cell lines	Oncogene	Diagnosis
Roscigno [159]	2020	Breast cancer	TLR4	T47D and MDA-MB-MB-231cell lines	Tumor suppressor	Diagnosis
Wang [160]	2018	Renal cell carcinoma	TRL4	27 T 27N tissues 786-O, ACHN, Caki-1, A498, GRC-1 and OS-RC-2 cell lines BALB/c nude mice	Tumor suppressor	Diagnosis
Wang [162]	2018	Colorectal cancer	COX-2/ALOX5	42 T 42N tissues HT29, HCT15, SW480 and SW1116 cell lines	Tumor suppressor	Diagnosis and prognosis
Liu [168]	2018	Melanoma	HK2	86 T tissues HEK293T cell line BALB/c nude mice	Tumor suppressor	Diagnosis and prognosis
Pang [171]	2021	Non-small cell lung cancer	RAP2B	35 T 35N tissues A549 and NCI-H1299 cell lines BALB/C nude mice	Tumor suppressor	Diagnosis and prognosis
Li [174]	2022	Cervical cancer	CDC42	31 T 31N tissues SiHa, HeLa, and 293 T cell lines BALB/c nude mice	Tumor suppressor	Diagnosis and prognosis
Zhang [180]	2019	Breast cancer	PAK2	50 T 50N tissues BC MCF-7 cell line	Tumor suppressor	Diagnosis
Cui [185]	2019	Breast cancer	PKCα	10 T 10N tissues MCF-7, MD-MB231, MDA-MB-468, and SK-BR3 cell lines	Tumor suppressor	Diagnosis
Wang [191]	2020	Lung adenocarcinoma	COPB2	H1299, A549, SK-MES-1, NCI-H23, and H1975 cell lines	Tumor suppressor	Diagnosis
Peng [194]	2020	Glioma	AQP4	50 T 50N tissues U251, A172, T98G, HS683, and U138 cell lines BALB/c nude mice	Tumor suppressor	Diagnosis
Wang [201]	2020	Pancreatic cancer	TSPAN1	PANC-1, BxPC3, and ASPC1 cell lines	Tumor suppressor	Diagnosis
Sun [206]	2020	Esophageal cancer	KIAA0101	83 T 83N tissues EC9706, EC109, KYSE150, KYSE450, TE1, and TE10 cell lines	Tumor suppressor	Diagnosis and prognosis

## JAK/STAT and MAPK signaling pathways

**Fig. 1:**
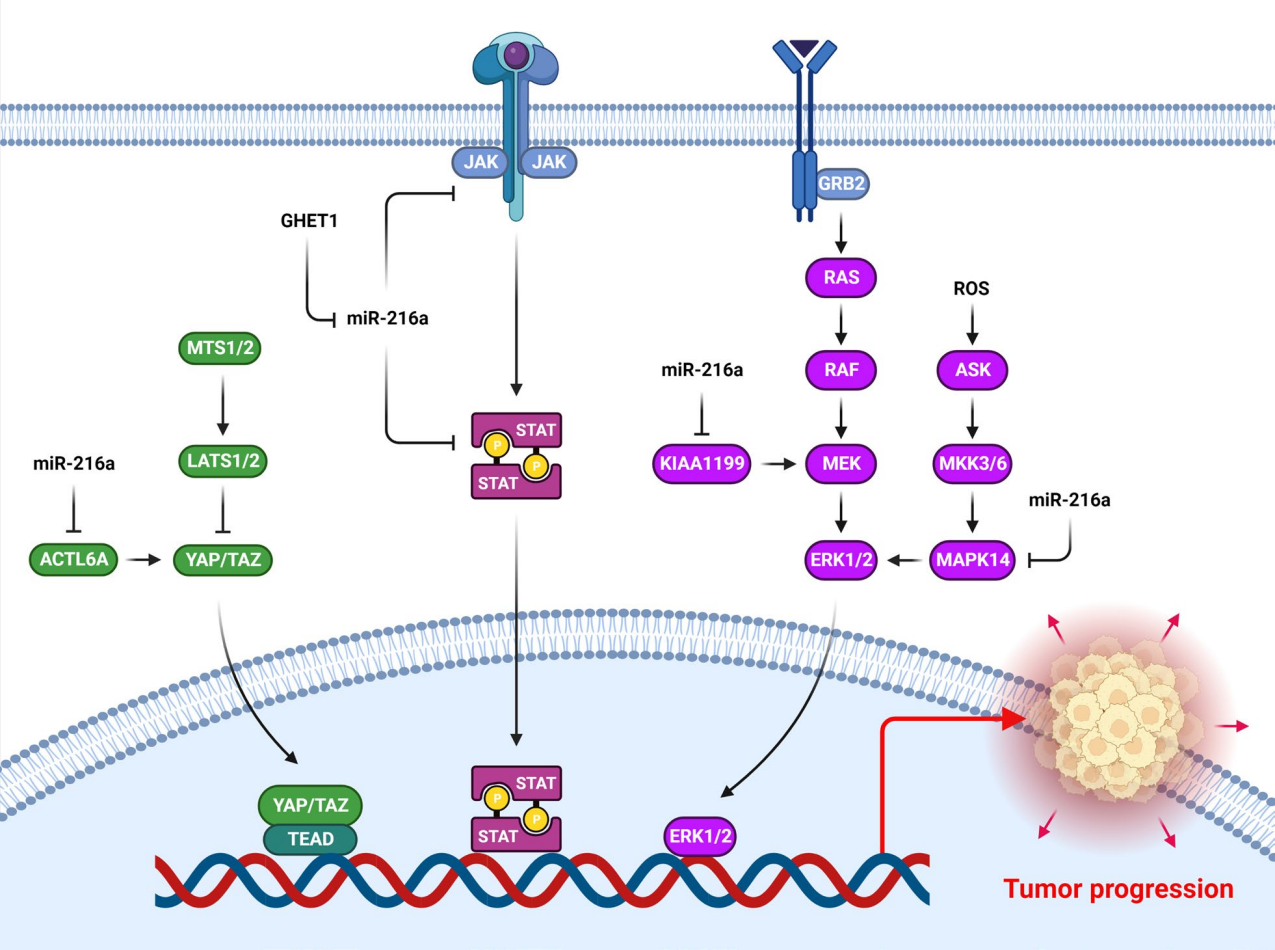
Role of miR-216a during tumor progression by regulation of JAK/STAT, MAPK, and Hippo signaling pathways. (Created with BioRender.com)

Cytokines, interleukins, and growth factors, lead to the activation of the JAK/STAT signaling pathway. Association of Cytokines with their correlative trans-membrane receptor subunits, causing multimerization with other subunits and conformational change in the receptor complex [18, 19]. JAK2 belongs to the Janus Kinases family of protein tyrosine kinases that plays an important role during tumor progression through STAT3 phosphorylation [20, 21]. The JAK2/STAT3 cascade plays a key role in many cellular processes, including growth, division, programmed cell death, immunological escape and resistance, and tumor angiogenesis [22, 23]. STAT3 is an inactive monomeric transcription factor in the cytoplasm. This transcription factor is dimerized and translocated into the nucleus after being phosphorylated by JAK2 to activate the target genes [24, 25]. STAT3 triggers cellular transformation and facilitates tumor initiation and progression by regulation of c-Myc, Bcl-xL, CCND1, and VEGF [26]. It has been shown that miR-216a has a key role during tumor progression by regulation of JAK/STAT signaling pathway (Fig. 1). MiR-216a significantly suppressed cell proliferation while induced programmed cell death in pancreatic tumor cells by inhibiting JAK2. MiR-216a also suppressed STAT3 phosphorylation, which resulted in the down regulation of anti-apoptotic genes such as survivin and XIAP [27]. MiR-216a reduced pancreatic tumor growth via JAK2 targeting [28]. STAT3 up regulated the miR-216a that targeted PTEN. Suppression of miR-216a reduced the cisplatin resistance in ovarian tumor cells [29]. Long noncoding RNAs (lncRNAs) are promising therapeutic targets and diagnostic factors in a variety of disorders [30, 31]. They are involved in biological processes such as chromatin remodeling, transcriptional activation, and chromosomal inactivation [32]. LncRNAs mainly act as competing endogenous RNAs (ceRNAs), which compete for miRNAs to control various mRNA transcripts [33]. GHET1 enhanced the glioma cell invasion by miR-216a down regulation that stimulated the JAK2/STAT3 and p53/survivin signaling pathways [34]. MiR-216a was considerably down regulated in GC tissues as compared to corresponding healthy tissues that was associated with poor prognosis. MiR-216a inhibited JAK2/STAT3 cascade as well as the expression of downstream targets such as Slug, Snail, and Twist in GC cells [35].

The MAPK signaling plays an important role in cell biology and functions through receptor tyrosine kinases (RTKs) that activate the RAF/MEK/ERK axis [36, 37]. Activated ERKs are accumulated in the nucleus or remain in the cytoplasm, where they can phosphorylate several substrates that modulate cell activities [38]. Sorafenib acts as a tyrosine kinase suppressor with multiple targets. It can inhibit tumor cell proliferation by suppressing the RAF/MEK/ERK cascade as well as many other signaling pathways. It can also suppress the VEGF and PDGF receptors, hence preventing tumor angiogenesis [39]. MAPK14 activation has a crucial function in drug resistance in hepatocellular carcinoma (HCC) [40]. MiR-216a has a key role during tumor progression by regulation of MAPK signaling pathway (Fig. 1). There was significant MAPK14 up regulation in sorafenib resistant HCC tumors. MiR-216a-3p increased sorafenib response in xenograft HCC tumor nude mice models by targeting MAPK14 and suppressing the MEK/ERK and ATF signaling cascades [41]. KIAA1199 elevates cytosolic calcium through facilitating endoplasmic reticulum (ER) calcium leakage, which subsequently stimulates the PKCa-MEK1/2-ENK1/2 axis [42]. KIAA1199 promoted EGF-induced EMT by EGFR stability and phosphorylation of MEK1, and ERK1/2 in cervical tumor cells [43]. Under expression of KIAA1199 reduced CRC cell migration and invasion. MiR-216a inhibited CRC invasion by KIAA1199 targeting. KIAA1199 was significantly correlated with poor prognosis [44].

## PI3K/AKT and TGF-β signaling pathways

**Fig. 2:**
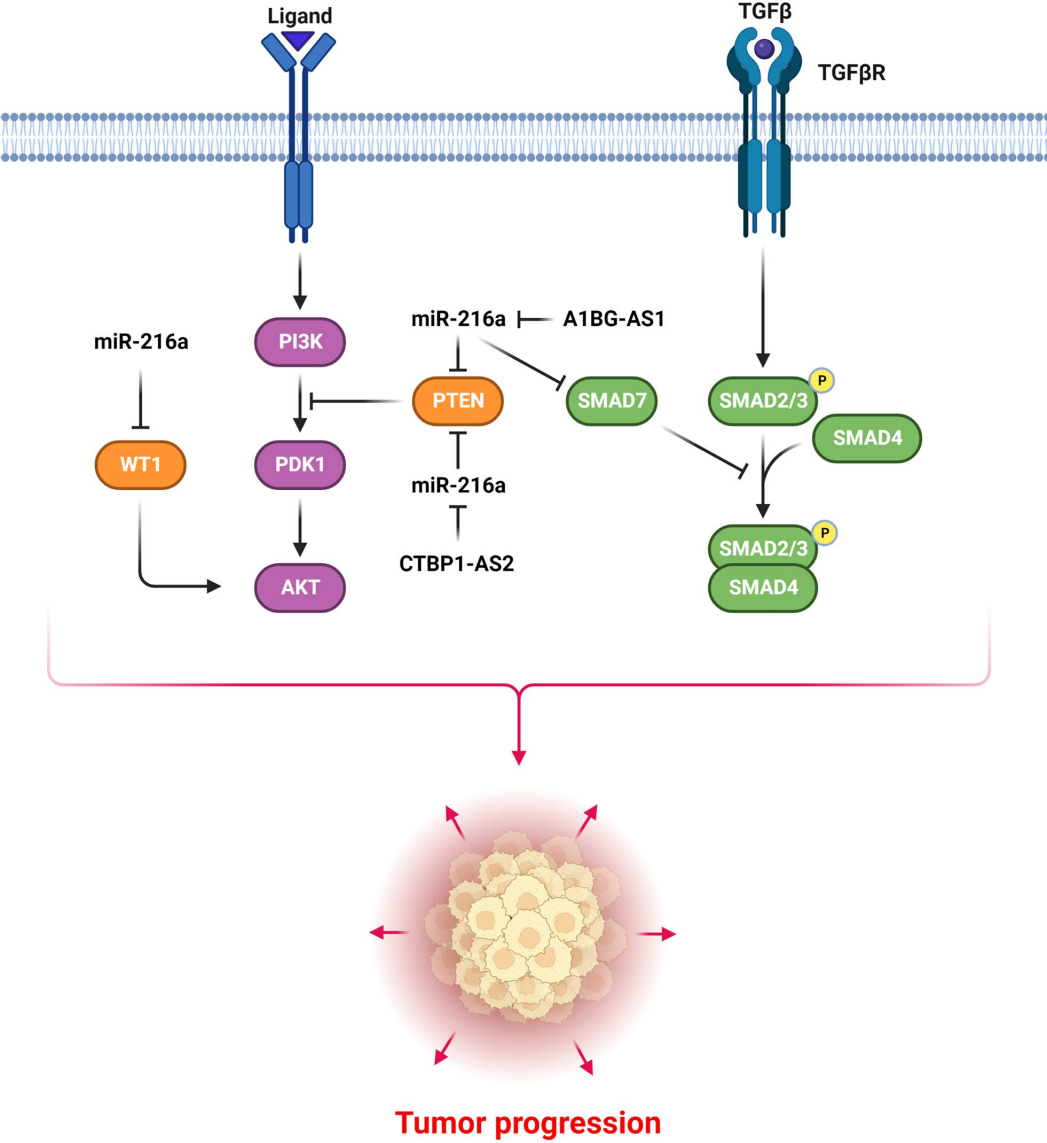
Role of miR-216a during tumor progression by regulation of PI3K/AKT and TGF-β signaling pathways. (Created with BioRender.com)

PI3K/Akt pathway is known as one of the most critical pathways in modulating cell survival and proliferation [45]. PI3K activates the AKT that induces cell proliferation by CCND1 up regulation [46]. PTEN inhibits the growth and dissemination of HCC cells as a negative regulator of PI3K/AKT pathway [47]. MiR-216a has a key role during tumor progression by regulation of PI3K/AKT signaling pathway (Fig. 2). Smad7 acts as a tumor suppressor in HCC by inhibiting cell growth while triggering programmed cell death [48]. The A1BG antisense RNA 1 (A1BG-AS1) was down regulated in HCC. It inhibited HCC cell growth, metastasis, and invasion by miR-216a-5p sponging and PTEN and Smad7 up regulations [49]. There was CTBP1-AS2 down regulation in ovarian cancer (OC). CTBP1-AS2 inhibited the OC cell proliferation by miR-216a sponging and subsequent PTEN up regulation [50]. There was significant miR-216a up regulation in ovarian cancer tissues and cells that promoted cell proliferation and invasion by inhibiting PTEN [51]. WT1 is an oncogene that has been identified to be overexpressed in a variety of solid tumors and blood cancers, making it a prospective therapeutic target for cancer treatment [52]. Overexpression of miR-216a or knockdown of KRT7 inhibited PI3K and AKT phosphorylation in PC cells, whereas WT1 stimulated the PI3K/AKT signaling cascade. Therefore, miR-216a regulated the WT1/KRT7 axis and inhibited the PI3K/AKT pathway to prevent PC progression [53].

TGF-β as a growth factor is implicated in the modulation of cell growth, autophagy, apoptosis, and EMT [54]. It principally participates in different biological processes in the body via two pathways: the classic SMAD-associated pathway and the non-SMAD-associated pathway. TGF-β receptors mediate the SMAD-related classical pathway [55, 56]. The association between TGF-β and TβRII can stimulate the kinase activity of TβRI and promotes the phosphorylation of TβRI. Consequently, activated TβRI could phosphorylate downstream SMAD proteins. These activated SMAD proteins could interact with the chaperone protein SMAD4 and translocated to the nucleus and modulate the expression of TGF-β target genes [57]. MiR-216a has a pivotal role during tumor progression by regulation of TGF-β signaling pathway (Fig. 2). Epithelial-mesenchymal transition (EMT) is a normal developmental process involved in tumor invasion in which epithelial cells transform into mesenchymal cells. Vimentin is overexpressed while cell adhesion molecules such as E-cadherin are under expressed during EMT [58]. As a member of the SMAD family of proteins, SMAD7 is a TGF-β superfamily ligand. By analyzing miRNA expression profiles in patients with HCC tissues with early-recurrent and non-recurrent HCC, researchers discovered that early HCC recurrent disease was correlated with miR-216a up regulation. MiR-216a positively regulated TGF-β and the canonical pathway implicated in the promotion of the PI3K/Akt cascade in HCC cells by inhibiting SMAD7 and PTEN, resulting in tumor relapse and sorafenib resistance [59]. There was HOTTIP up regulation in prostate cancer (PCa) tissues that was correlated with larger tumor size and a higher TNM stage. HOTTIP inhibition down regulated the Vimentin and Snai1, while up regulated the CDH1. HOTTIP enhanced the growth and metastasis of PCa cells by miR-216a-5p sponging [60].

## Developmental signaling pathways

Wnt is a pivotal signaling pathway for tissue morphogenesis and regeneration that is activated by the canonical or non-canonical pathways [61]. The activation of the canonical pathway occurs in the presence of Wnt ligands. Wnt ligands could interact with the Frizzled (Fz) receptor and LRP5/6 co-receptor that finally stabilizes the cytoplasmic β-catenin [62, 63]. β-catenin is translocated to the nucleus where it is associated with LEF/TCF family members to regulate the WNT target genes [64, 65]. DANCR silencing has been shown to diminish cell migration, survival, and stem-like properties. DANCR increased β-catenin expression, which was then inhibited by miR-216a in non-small-cell lung cancer (NSCLC) cells. DANCR promoted NSCLC stemness and chemo resistance by activating Wnt and Sox2 [66]. LGR5 as an orphan G protein-coupled receptor (GPCR) is involved in developmental processes [67, 68]. It regulates Wnt signaling cascade via interacting with its associated ligand R-spondin and mediates the accumulation of nuclear β-catenin. LGR5 exerts as a stem cell factor and promotes the maintenance of cancer stem cells, self-renewal, and stem cell proliferation by activation of downstream Wnt/β-catenin-signaling cascade [69]. It has been indicated that LGR5 could induce cell mobility, invasion, tumorigenesis, and EMT in cancer cells through activation of the Wnt/β-catenin pathway [70]. MiR-216a markedly inhibited glioma cell growth and invasion by inhibiting LGR5 [71].

The Sonic hedgehog (Shh) is also another developmental signaling pathway that has key roles in tumor cell growth and differentiation. It can be activated through the interaction of Shh with the cell surface receptor Patched (PTCH) that leads to the phosphorylation of the SMO receptor [72]. The association between Hh ligands and PTCH induces GLI transcription factors [73]. The GLI proteins translocate into the nucleus, where they promote the target genes expression and also induce cell growth, survival, and differentiation [73]. Tectonic family member 1 (TCTN1) is a member of the tectonic trans-membrane protein family that is implicated in the Hedgehog (Hh) signaling pathway [74]. Bcl-2 is a negative regulator of apoptosis that is located in inner mitochondrial membrane [75]. Bad is capable of triggering programmed cell death by suppressing Bcl-2 and Bcl-xL [76, 77]. TCTN1 knockdown was discovered to promote apoptosis in thyroid cancer cells via up regulation of CASP3 and PARP, while suppression of Bcl-2 [78]. Increased miR-216a-5p expression in ESCC cells was discovered to significantly inhibit cell growth by TCTN1 targeting. MiR-216a-5p suppressed cell proliferation by PCNA down regulation. Overexpression of miR-216a-5p in ESCC cells resulted in a significant reduction in PCNA and Bcl-2 expression levels while Bad up regulation. MiR-216a-5p repressed esophageal squamous cell carcinoma (ESCC) cell proliferation while promoted apoptosis via TCTN1 targeting [79].

Hippo signaling plays a vital role in tumor progression. Activation of the Hippo pathway leads to MST1/2 phosphorylation and stimulates LATS1/2, which can phosphorylate YAP/TAZ, causing the YAP/TAZ suppression [80]. The phosphorylation of LATS induces the cytoplasmic translocation of YAP proteins via association with 14-3-3 proteins [81, 82]. MiR-216a has a key role during tumor progression by regulation of Hippo signaling pathway (Fig. 1). Actin-like 6A (ACTL6A) is a component of the SWI/SNF that regulates chromatin remodeling, nuclear transition, and transcription regulation [83]. ACTL6A is overexpressed in progenitor and stem cells, and is involved in cell self-renewal [84, 85]. Yes-associated protein (YAP) is an essential member of Hippo pathway and plays a key role in the regulation of tissue homeostasis processes [86]. YAP is dephosphorylated in response to a variety of stimuli, and then it is transferred into the nucleus where it interacts with a transcriptional co-activator with a PDZ binding motif to increase the expression of the target gene [87]. MiR-216a-3p reduced the cervical tumor cell growth and invasion by inhibiting ACTL6A that subsequently enhanced YAP phosphorylation while reduced YAP/TAZ-mediated transcriptional activity [17].

## Transcription factors

**Fig. 3:**
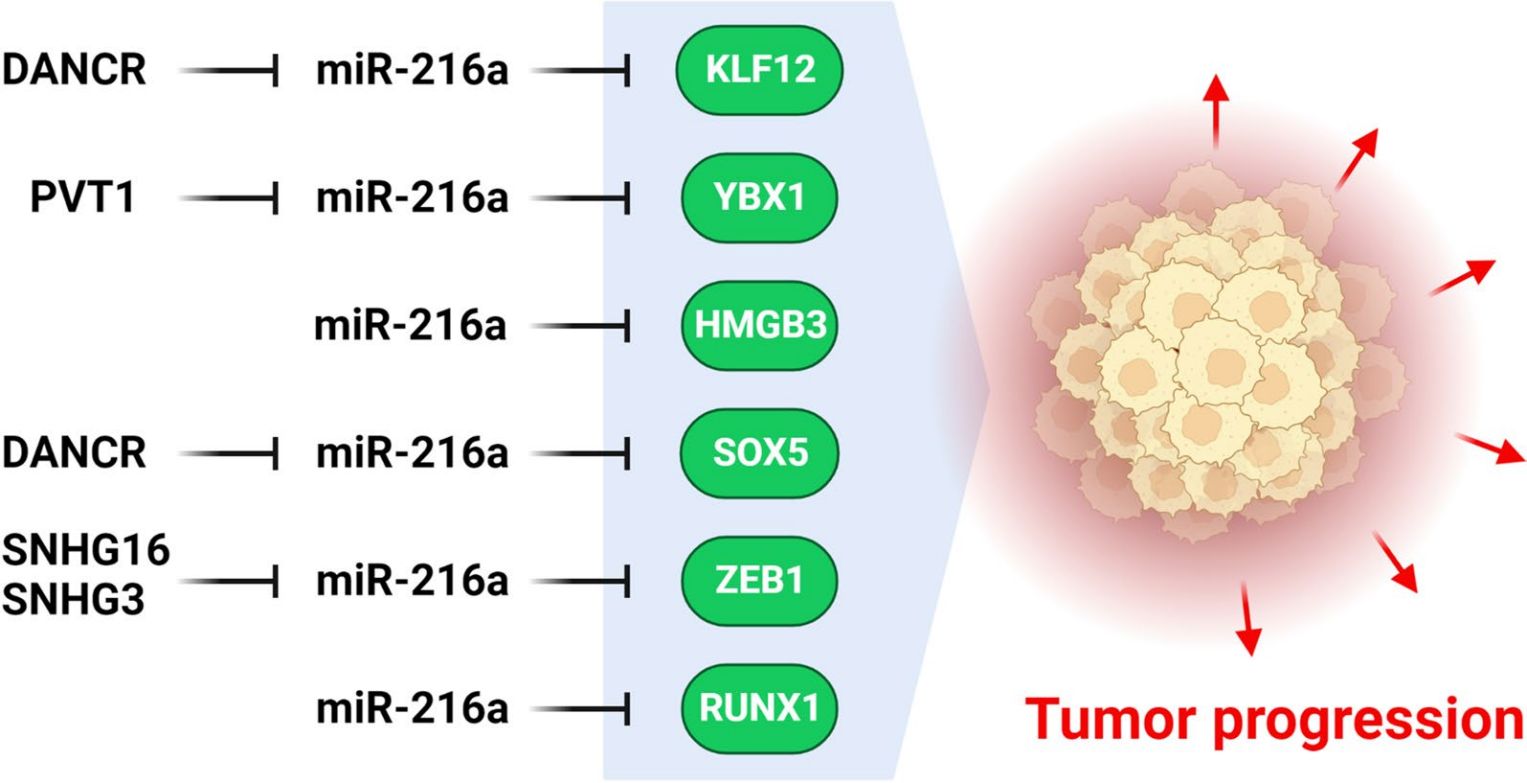
Role of miR-216a during tumor progression by regulation of transcription factors. (Created with BioRender.com)

Transcription factors are the key molecular targets for the miR-216a during tumor progression (Fig. 3). Y-box binding protein 1 (YBX1) belongs to the cold-shock protein superfamily that is involved in transcriptional and translational regulations [88, 89]. It has different pro-oncogenic roles in cancers, including tumor metastasis and chemotherapy resistance [90]. It has been demonstrated that phosphorylation of YBX1 through numerous kinases such as AKT, S6K, and RSK via receptor tyrosine kinase and integrin-associated kinase promotes nuclear transportation of YBX1 in different tissues with transcriptional activation of several genes containing drug resistance and tumor growth linked genes [91]. YB-1 expression was shown to be elevated in pancreatic cancer cells and tissue samples. It has anti-metastatic activity in pancreatic cancer and has been recognized as a target of miR-216a. MiR-216a reduced pancreatic tumor cell invasion by YB-1 targeting [92]. The MAPK/ERK cascade stimulates YBX1 and subsequently transfer it into the nucleus, promoting the development of B-cell lymphoma [93]. YBX1 is also involved in tumor progression via the PI3K/Akt/mTOR signaling cascade [94]. MiR-216a suppressed Diffuse Large B Cell Lymphoma (DLBCL) cell survival, growth, and invasion by targeting YBX1 [95]. There was miR-216a-5p down regulation in colorectal cancer (CRC) tissues that was correlated with poor prognosis. MiR-216a-5p suppressed CRC cell growth and invasion by inhibiting YBX1. PVT1 overexpression has been proposed to overturn the anti-tumor impact of miR-216a-5p on CRC cells. MiR-216a5p also caused CDH1 up regulation while CDH2, Vimentin, and Snail down regulations [96].

BRD4 enhances tumor progression and induces EMT tumor cells [97]. It induced the stemness characteristic of gastric cancer (GC) cells by MIR216A promoter methylation and subsequent miR-216a-3p down regulation. Wnt3a was found to be a direct downstream effector of miR-216a3p, implying that the Wnt cascade is required for the regulation of stemness features in GC cells via the BRD4/miR-216a-3p axis [98]. High mobility group box 3 (HMGB3) is involved in the regulation of self-renewal and cell differentiation [99]. It has an important regulatory role in cell growth and apoptosis and its deregulation can lead to malignant breast cancer [100–102]. MiR-216a hyper methylation led to HMGB3 overexpression via binding to the 3'UTR, which subsequently stimulated the Wnt/β-catenin signaling pathway and enhanced malignant growth and migration of esophageal tumor cells [103]. There were significant DANCR up regulation while miR-216a-5p down regulation in HCC cells. DANCR suppression reduced HCC cell growth and division through the miR-216a-5p/KLF12 axis [104]. There were significant DANCR up regulation while miR-216a-5p down regulation in Oral Squamous Cell Carcinoma (OSCC) tissues and cells that were associated with a higher TNM stage, lower differentiation level, and node metastasis. DANCR up regulated the KLF12 by functioning as a molecular sponge of miR-126-5p, facilitating OSCC metastasis and invasion [105].

Autophagy is a mechanism within the cell that eliminates and recycles defective organelles and proteins [106, 107]. SRY-related high-mobility-group box 5 (SOX5) is a developmental transcription factor that promotes tumor progression in a variety of cancers [108]. There was DANCR up regulation in osteosarcoma tissues that was positively correlated with the grade of tumor. DANCR inhibition suppressed osteosarcoma cell growth and invasion while induced apoptosis via miR-216a-5p/SOX5 axis [109]. There was DANCR up regulation in lung cancer tissues that was correlated with poor prognosis. DANCR promoted lung tumor cell invasion via miR-216a targeting [110].

Zinc finger E-box binding homeobox 1 (ZEB1) is also a critical mediator of EMT activation and self-renewal. ZEB1 could directly interact with the promoter regions of epithelial genes to inhibit their transcription and induce EMT through regulating the transcription of mesenchymal genes [111, 112]. ZEB-1 regulates the inhibition of CDH1 which promotes the EGFR/ERK axis in tumor cells [113]. There was SNHG16 up regulation in cervical cancer tissues that was correlated with advanced FIGO stage, larger tumor size, and lower differentiation. It was involved in cervical cancer progression by regulation of miR-216-5p/ZEB1 axis [114]. SNHG3 was found to be up regulated in NSCLC tissues and cells. SNHG3 inhibition reduced NSCLC cell growth and invasion while promoted apoptosis through miR-216a/ZEB1 axis [115].

RUNX1 is a transcription factor that has key role in hematopoiesis [116]. It reduces the tumor sphere formation and directly declines ZEB1 expression and also suppress the stem cell phenotype [117]. RUNX1 has been demonstrated to suppress NF-kB pathway by interacting with the IkB kinase. MiR-216a-3p may function as a tumor promoter in GC via inhibiting RUNX1 and stimulating the NF-kB signaling pathway. MiR-216a-3p was markedly up regulated in GC tissues that were associated with the prognosis. MiR-216a-3p significantly up regulated the CCND1, Bcl-2, MMP2, and MMP9 [118].

## Autophagy, apoptosis, and cell cycle regulation

**Fig. 4:**
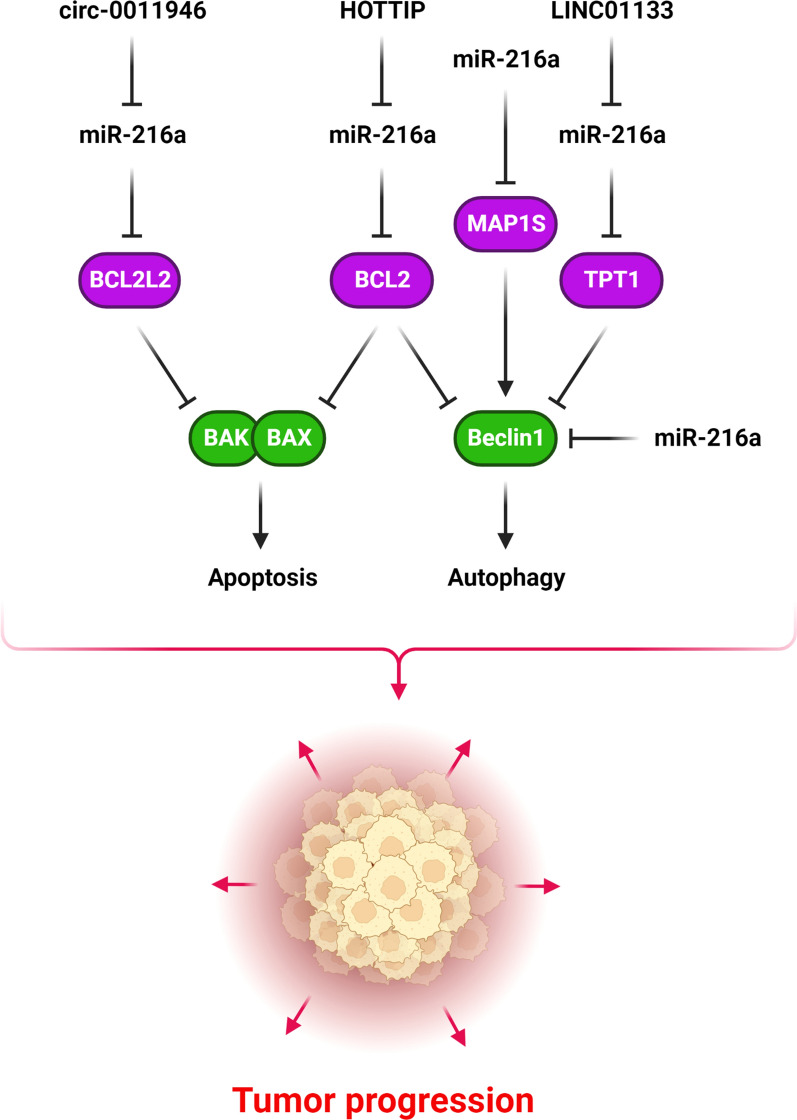
Role of miR-216a during tumor progression by regulation of apoptosis and autophagy. (Created with BioRender.com)

Autophagy is a catabolic process that degrades cytosolic proteins and organelles in response to cellular stress. This process is assumed to be the underlying cause of cancer cell radiation resistance [119]. In autophagy as a self-proteolytic cellular degradation mechanism, defective proteins and organelles are transported to lysosomes for destruction [120]. MiR-216a has a key role during tumor progression by regulation of autophagy and apoptosis (Fig. 4). This process removes highly toxic chemicals, preserves tissue homeostasis, and promotes cancer cell survival. Nevertheless, highly active autophagy results in apoptosis [121]. The production of autophagosomes is induced by class III phosphoinositide 3-kinase and beclin-1 during autophagy [122]. Beclin-1 is an autophagosome-forming factor that is up regulated in autophagy [123]. MiR-216a was discovered to markedly inhibit beclin-1 and autophagy processes in radio resistant pancreatic tumor cells, resulting in increased sensitivity to radiotherapy [124]. HOTTIP was strongly associated with GC recurrence in patients who received cisplatin treatment. HOTTIP increased cisplatin resistance and suppressed autophagy and apoptosis in GC cells through miR-216a-5p sponging and Bcl-2/Beclin1 axis regulation [125]. Microtubule associated protein 1S (MAP1S) plays a key regulatory role in promoting autophagy flux [126]. There is also a relationship between TGF-β/MAP1S-pathway-mediated autophagy and carcinogenesis inhibition [127]. There were miR-216a down regulations in CRC tissues and cells. MiR-216a inhibited autophagy by disrupting the TGF-β/MAP1S cascade in CRC cells [128]. Translationally controlled tumor protein (TCTP) is a highly conserved protein participated in cell proliferation and apoptosis [129]. It is also known as a modulator of tumor recurrence that reduces the expression level of p53 [130]. TPT1 has also been demonstrated to operate as a negative regulator of autophagy via BECN1 and the mTORC1-mediated pathway [131]. There was miR-216a-5p down regulation in pancreatic cancer (PC) tissues that was correlated with poor prognosis and increased tumor cell migration. MiR-216a-5p inhibited pancreatic tumor cell growth and motility by TPT1 targeting. LINC01133 was also reported to enhance PC cell growth, division, and migration via inhibiting miR-216a-5p [132]. B cell lymphoma-2-like 2 protein (BCL2L2) is a member of the BCL2 family that plays a crucial role in human malignancies [133]. BCL2L2 enhances tumor progression by facilitating cell growth and division [134]. Circ-0011946 inhibition reduced OSCC cell growth and metastasis while induced apoptosis via the miR-216a-5p/BCL2L2 axis [135]. HOTTIP induced chemo resistance in small cell lung cancer through the miR-216a/BCL-2 axis [136]. MiR-216a-5p reduced cell growth, division, and metastasis in lung cancer through regulating Bcl-2/Bax/Bad protein expression [137].

Cyclin-dependent kinases (CDKs) are a group of cell cycle related kinases that have important regulatory functions during cell cycle progression [138]. CDKs are important regulators of cell cycle progression and have been proposed as potential therapeutic targets for cancer therapy [139]. CDK14 is an important cell cycle regulator by interacting with CCND3 [140, 141]. It promotes Wnt signaling by mediating LRP6 phosphorylation [140, 142]. CDK14 silencing down regulated PI3K and inhibited AKT phosphorylation in pancreatic cancer cells [143]. CDK14 was discovered to be associated with overall survival and prognosis in osteosarcoma patients. Patients with overexpressed miR-216a showed improved overall survival, implying that miR-216a plays a predictive and prognostic function in osteosarcoma. MiR-216a inhibited osteosarcoma cell growth and invasion by down regulating CDK14. The miR-216a/CDK14 axis promoted Wnt pathway in osteosarcoma cells via modulating LRP6 phosphorylation and Wnt downstream genes. MiR-216a/CDK14 axis was also essential in the phosphorylation of PI3K and Akt in osteosarcoma cells. MiR-216a down regulated CDH2 while up regulated CDH1 via CDK14 targeting in osteosarcoma [144]. BTG2 as a member of the TOB/BTG gene family is involved in G1/S cell cycle progression [145, 146]. BTG2 negatively mediates CCND1 and reduces the expression level of FoxM1 via suppressing the association of CCNB1/CDKs [147]. CircFLNA reduced the bladder tumor growth via miR-216a-3p/BTG2 axis [148].

## Structural factors

**Fig. 5:**
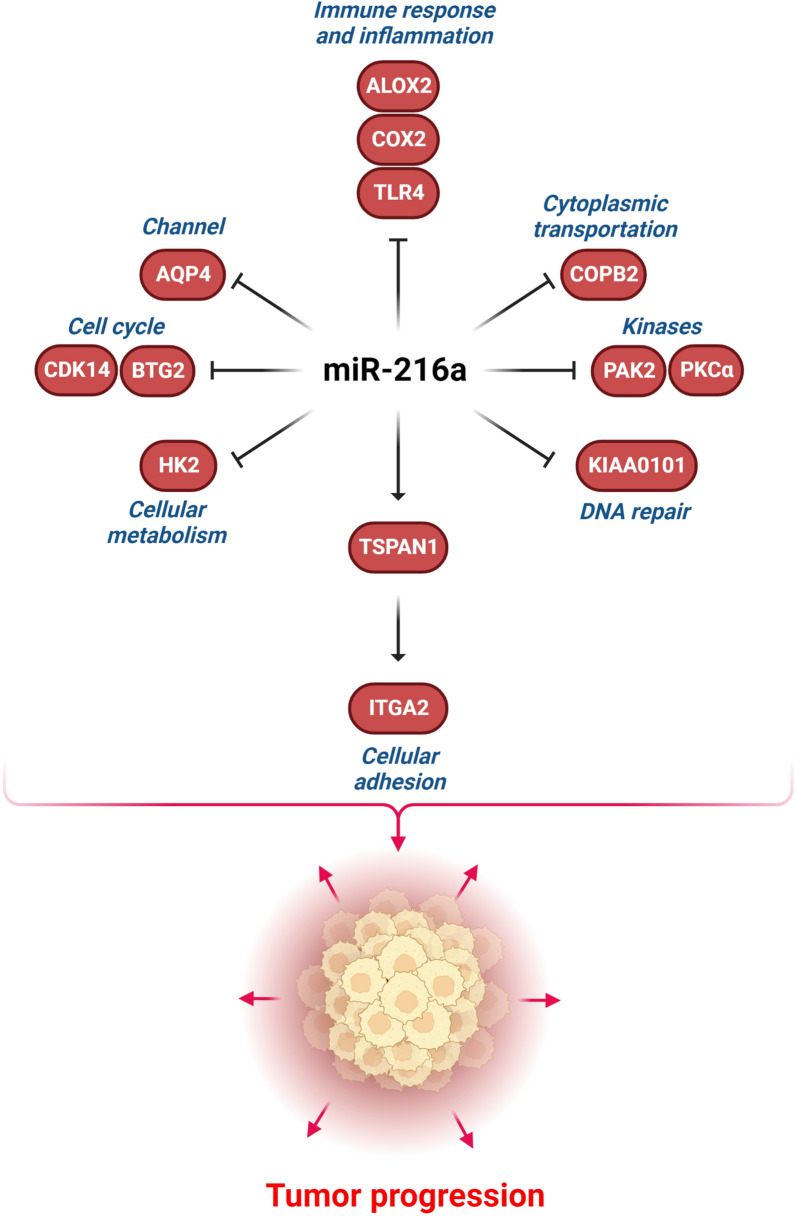
Role of miR-216a during tumor progression by regulation of structural proteins involved in cell adhesion, metabolism, DNA repair, and immune response. (Created with BioRender.com)

Various structural proteins involved in immune response, cell adhesion, cellular metabolism, and DNA repair can also be targeted by miR-216a during tumor progression (Fig. 5). The tumor microenvironment plays a key role in the modulation of oncogenic events through macrophages, neutrophils, mast cells, T/B lymphocytes, and also stromal cells [149]. There are three types of interactions between tumor microenvironment components as well as between these components and tumor cells, including direct contact, paracrine, or autocrine signaling [150, 151]. Cancer-associated fibroblasts (CAFs) constitute the majority of tumor stroma [152]. CAFs secrete inflammatory cytokines, which results in the stimulation of pathways that promote tumor cell growth and self-renewal preservation [153]. Toll-like receptors (TLRs) are a class of cell surface recognition receptors that form a connection between the tumor microenvironment and tumor cells. They are not only implicated in the defense against pathogen attack, but they can also enhance tumor cell proliferation [154]. TLR4 activation causes a pro-inflammatory response, which results in the synthesis and release of cytokines such as IL-6 and IL-8 [155, 156]. TLR4 is involved in tumor cell adhesion and invasion in a variety of human malignancies [157, 158]. MiR-216a-5p functioned as an inhibitor of breast tumor progression and promoted the secretion of IL-6 pro-inflammatory cytokine by TLR4 targeting [159]. There was significant miR-216a down regulation in renal cell carcinoma (RCC) tissues. It reduced RCC cell growth and invasion, while induced apoptosis via TLR4 targeting [160].

COX and 5-lipoxygenase (ALOX5) play a key role in the synthesis of prostaglandins and leukotrienes, respectively. These were first recognized as being essential in the regulation of inflammation. Anti-inflammatory drugs, such as COX2 suppressors are conventional drugs used in the treatment of breast cancer [161]. Although, ALOX5 and COX2 play their roles via different cellular pathways, they have comparable mechanisms for modulating cell survival. MiR-216a-3p suppressed CRC cell growth by negatively modulating the expression of COX2 and ALOX5 in CRC cells. CRC patients with T3 and T4 stages had significantly higher levels of COX2 and ALOX5 expressions compared to healthy tissues. COX2/ALOX5 up regulation was significantly correlated with poor prognosis. MiR-216a-3p inhibited CRC cell growth by suppressing COX2 and ALOX2 [162].

Aerobic glycolysis, which exhibits aberrant metabolism defined by excessive glycolysis despite the presence of sufficient oxygen, is recognized as a typical characteristic of tumor cells .[163, 164] This process increases the lactate synthesis and glucose uptake, which stimulates tumor growth. Hexokinase 2 (HK2) as the first enzyme in glycolysis catalyzes the glucose-6-phosphate production [165]. HK2 up regulation has been reported in numerous malignancies and promotes tumor growth by the glycolysis induction [166, 167]. MiR-216a-5p has been discovered to reduce the glycolysis and cell growth by HK2 targeting in uveal melanoma cancer cells [168].

RAP2B belongs to the Ras superfamily that is involved in the regulation of cell proliferation and migration [169, 170]. CCAT1 promoted NSCLC proliferation while reduced apoptosis via the miR-216a-5p/RAP2B axis [171]. CDC42 is a component of the Rho GTPase family and is involved in cell proliferation and migration [172, 173]. HCP5 promoted the cervical tumor initiation and progression via the miR-216a-5p/CDC42 axis [174].

PAK2 is a kinase involved in a variety of intracellular processes, including cytoskeletal remodeling and cell migration [175, 176]. The Rac and CDC42 stimulate PAK2 [175, 177]. The size and prognosis of malignant tumors have been correlated with PAK2 activation [178, 179]. MiR-216a-5p reduced breast tumor cell growth and invasion via PAK2 targeting [180]. Protein kinase C alpha (PKCα) is a member of the PKC family [181]. PKCα expression is contributed with poor prognosis in ER-positive breast cancers [182, 183]. It promotes breast tumor cell migration via FOXC2-mediated inhibition of p120-catenin [184]. There was significant miR-216a down regulation in breast cancer cells. It promoted the breast tumor cell apoptosis via PKCα targeting [185].

The coatomer protein complex subunit beta 2 (COPB2) plays a vital role in intracellular transportation by forming transport vesicles [186]. It has been indicated that COPB2 participates in the modulation of extracellular membrane transportation and stimulation of retrograde transport between the Golgi complex and ER [187]. COPB2 can mediate the growth and apoptosis of cancer cells by activating the RTKs- and JNK/c-Jun-signaling cascades [188]. Under expression of COPB2 induces tumor cell apoptosis [189, 190]. COPB2 inhibition significantly up regulated the CDH1 while down regulated the CDH2 and Vimentin that reduced lung tumor cell invasion. MiR-216a-3p reduced lung tumor cell invasion while promoted the apoptosis by COPB2 targeting [190, 191].

Aquaporin-4 (AQP4) is a critical molecule in the central nervous system that participated in preserving water and ion homeostasis and has been indicated to play a key role in tumor cell invasion [192]. AQP4 is also associated with α-syntrophin which interacts with the actin cytoskeleton and β-dystroglycan. Therefore, AQP4 can be involved in modification of the cellular cytoskeleton [193]. There were LINC00461 up regulations in glioma tissues and cells. LINC00461 silencing inhibited glioma cell growth, invasion, and temozolomide (TMZ) tolerance via miR-216a/ AQP4 axis [194].

Tetraspanin 1 (TSPAN1) is a small trans-membrane protein engaged in cell migration and proliferation [195, 196]. Integrins as the cell adhesion receptors, directly bind to diverse extracellular matrix (ECM) molecules and regulate cell growth, apoptosis, and invasion [197]. Deregulation of integrin is associated with tumor progression by disrupting the cell migration [198]. Integrin alpha 2 (ITGA2) is a trans-membrane receptor that facilitates cell adherence to the ECM that is deregulated in various tumor types [199, 200]. TSPAN1 has the ability to modulate methylation-related enzymes and thereby influence the methylation level of the ITGA2 promoter. TSPAN1 up regulated TET2 while down regulated DNMT3B and DNMT1. TSPAN1 regulated methyltransferases that resulted in ITGA2 hypo methylation in PC. MiR-216a/TSPAN1/ITGA2 axis was implicated in the regulation of PC progression [201].

KIAA0101 or proliferation cell nuclear antigen (PCNA) protein is implicated in the modulation of DNA repair and cell proliferation, cell cycle development, and migration [202]. It preserve cells from UV-associated cell death [203]. Down regulation of KIAA0101 suppresses tumor cell progression and glycolysis by inactivating the PI3K/AKT/mTOR pathway [204]. The KIAA0101 protein has been deregulated in multiple malignancies that were associated with poor prognosis [202, 205]. There was miR-216a-5p down regulation in ESCC tissues that was correlated with poor prognosis. MiR-216a-5p suppressed ESCC cell growth and invasion by KIAA0101 targeting [206].

## Conclusions

Considering the importance of identifying non-invasive markers to facilitate early tumor detection, in the present review we investigated the role of miR-216a during tumor progression. It has been reported that miR-216a has mainly a tumor suppressor function through the regulation of signaling pathways and transcription factors, which ultimately changes the cell cycle, apoptosis, and autophagy. This study can be an effective step towards introducing the miR-216a as a non-invasive marker in tumor detection and treatment. MiRNA-based cancer therapy is designed based on the miRNA function inside the tumor cells by the inhibition of oncogenic miRNAs or induction of tumor suppressor miRNAs. However, there are some challenges to use the miRNAs in tumor targeted therapy including the miRNA degradation by the cytoplasmic nucleases and the adverse influences of the selected miRNAs in normal biological cellular functions. Therefore, the side effects can be expected following the miRNA targeted therapy. Optimization of the site specific and delivery methods can reduce the optimal antagomiRs or mimics concentrations that finally reduces the probable side effects of miRNA-based therapies in cancer patients. Since, miR-216a has mainly a tumor suppressive function in different tumor types, miR-216a mimics can be used as a method of choice in cancer patients. However, it is required to perform the in-vitro and animal studies to confirm the miR-216a as an efficient candidate for the targeted therapy in clinics. Moreover, assessment of the circulating miR-216a levels in different cancers is required to suggest that as a reliable non-invasive diagnostic marker in cancer patients.

## Data Availability

The datasets used and/or analyzed during the current study are available from the corresponding author on reasonable request.

## Abbreviations

A1BG-AS1: A1BG antisense RNA 1
ACTL6A: Actin-like 6A
AQP4: Aquaporin-4
BCL2L2: B cell lymphoma-2-like 2 protein
CAFs: Cancer-associated fibroblasts
COPB2: Coatomer protein complex subunit beta 2
CRC: Colorectal cancer
ceRNAs: Competing endogenous RNAs
CDKs: Cyclin-dependent kinases
DLBCL: Diffuse Large B Cell Lymphoma
ER: Endoplasmic reticulum
ECM: Extracellular matrix
Fz: Frizzled
GPCR: G protein-coupled receptor
GC: Gastric cancer
Hh: Hedgehog
HCC: Hepatocellular carcinoma
HK2: Hexokinase 2
HMGB3: High mobility group box 3
ITGA2: Integrin alpha 2
lncRNAs: Long noncoding RNAs
miRNAs: MicroRNAs
MAP1S: Microtubule associated protein 1S
NSCLC: Non-small-cell lung cancer
OSCC: Oral Squamous Cell Carcinoma
OC: Ovarian cancer
PC: Pancreatic cancer
PTCH: Patched
PCNA: Proliferation cell nuclear antigen
PKCα: Protein kinase C alpha
RTKs: Receptor tyrosine kinases
RCC: Renal cell carcinoma
Shh: Sonic hedgehog
SOX5: SRY-related high-mobility-group box 5
TCTN1: Tectonic family member 1
TMZ: Temozolomide
TSPAN1: Tetraspanin 1
TLRs: Toll-like receptors
TCTP: Translationally controlled tumor protein
YBX1: Y-box binding protein 1
YAP: Yes-associated protein
ZEB1: Zinc finger E-box binding homeobox 1
